# Association of HDL-Related Loci with Age-Related Macular Degeneration and Plasma Lutein and Zeaxanthin: the Alienor Study

**DOI:** 10.1371/journal.pone.0079848

**Published:** 2013-11-06

**Authors:** Bénédicte M. J. Merle, Cécilia Maubaret, Jean-François Korobelnik, Marie-Noëlle Delyfer, Marie-Bénédicte Rougier, Jean-Charles Lambert, Philippe Amouyel, Florence Malet, Mélanie Le Goff, Jean-François Dartigues, Pascale Barberger-Gateau, Cécile Delcourt

**Affiliations:** 1 INSERM, (Institut National de la Santé et de la Recherche Médicale), ISPED (Institut de Santé Publique d’Épidémiologie et de Développement), Centre INSERM U897-Epidemiologie-Biostatistique, Bordeaux, France; 2 Université de Bordeaux, Bordeaux, France; 3 Centre Hospitalier Universitaire (CHU) de Bordeaux, Service d’Ophtalmologie, Bordeaux, France; 4 INSERM, (Institut National de la Santé Et de la Recherche Médicale), U744, Lille, France; 5 Institut Pasteur de Lille, Lille, France; 6 Université Lille Nord de France, Lille, France; University of Florida, United States of America

## Abstract

**Background:**

Several genes implicated in high-density lipoprotein (HDL) metabolism have been reported to be associated with age-related macular degeneration (AMD). Furthermore, HDL transport the two carotenoids, lutein and zeaxanthin, which are highly suspected to play a key-role in the protection against AMD. The objective is to confirm the associations of HDL-related loci with AMD and to assess their associations with plasma lutein and zeaxanthin concentrations.

**Methods:**

Alienor study is a prospective population-based study on nutrition and age-related eye diseases performed in 963 elderly residents of Bordeaux, France. AMD was graded according to the international classification, from non-mydriatic colour retinal photographs. Plasma lutein and zeaxanthin were determined by normal-phase high-performance liquid chromatography. The following polymorphisms were studied: rs493258 and rs10468017 (*LIPC*), rs3764261 (*CETP*), rs12678919 (*LPL*) and rs1883025 (*ABCA1*).

**Results:**

After multivariate adjustment, the TT genotype of the *LIPC* rs493258 variant was significantly associated with a reduced risk for early and late AMD (OR=0.64, 95%CI: 0.41-0.99; p=0.049 and OR=0.26, 95%CI: 0.08-0.85; p=0.03, respectively), and with higher plasma zeaxanthin concentrations (p=0.03), while plasma lipids were not significantly different according to this SNP. Besides, the *LPL* variant was associated with early AMD (OR=0.67, 95%CI: 0.45-1.00; p=0.05) and both with plasma lipids and plasma lutein (p=0.047). Associations of *LIPC* rs10468017, *CETP* and *ABCA1* polymorphisms with AMD did not reach statistical significance.

**Conclusion:**

These findings suggest that *LIPC* and *LPL* genes could both modify the risk for AMD and the metabolism of lutein and zeaxanthin.

## Introduction

Age-related macular degeneration (AMD) is a degenerative disease of the central part of the retina (macula), responsible for half of the cases of blindness in industrialized countries [1]. This disease affects 2.5 million subjects in Europe [2] and 1.75 million in the USA [3]. It comprises two late forms both associated with severe visual impairment (neovascular and atrophic AMD), generally preceded by early, asymptomatic, retinal abnormalities (drusen, pigmentary abnormalities). It is a multifactorial disorder involving genetic and environmental factors [4]. Since 2005, major associations with genes in the complement pathway (complement factors H, B, C2 and C3) and the *ARMS2* gene have been identified [4]. Besides, the influence of environmental factors (in particular smoking and diet) has been highlighted by epidemiological studies [5–8]. In particular, lutein and zeaxanthin, two carotenoids of dietary origin, are suspected to play a key-role in the prevention against AMD [9]. 

Indeed, lutein and zeaxanthin are highly concentrated in the macula (30 to 10 000 times higher than in others tissues), in which they constitute the macular pigment (MP). MP may help protect against AMD because of its physical property of blue light filtration and its local antioxidant activity [9,10]. Epidemiological studies have reported a decreased risk for AMD in subjects with high plasma concentration or dietary intake of lutein and zeaxanthin [11–14]. The metabolism of these lipophilic molecules is closely interlinked with cholesterol metabolism [9]. Indeed, they are mainly carried by HDL [15]. In addition, several lipid transporters, such as SR-BI, CD36 or NCP1L1, are implicated in the transport of carotenoids [16]. Genetic variations in the corresponding genes of these transporters have been reported to be associated with alterations of plasma carotenoid status [16–18]. Alterations in cholesterol metabolism might therefore influence MP accumulation, with consequences on the risk for AMD. 

Associations of AMD with several genes implicated in HDL metabolism have been reported recently. In 2010, two genome-wide association studies [19,20] (GWAS) reported associations of AMD with the *hepatic lipase* (*LIPC*) gene, and suggested further associations with the *cholesterylester transfer protein* (*CETP*) gene, the lipoprotein lipase (LPL) gene and the *ATP-binding cassette transporter A1* (*ABCA1*) gene. These findings have been partly replicated in a few studies (Table 1) [21–31].

**Table 1:** Summary of studies about AMD and genes implicated in cholesterol metabolism.

**Study (First author, Year)**	**Design**	**Ethnicity**	**Subjects (n, AMD definition)**	**Associations with AMD (OR (95% CI)) OR for minor allele**				
				***LIPC*** rs10468017	***LIPC*** rs493258	***ABCA1*** rs1883025	***CETP*** rs3764261	***LPL*** rs12678919
			Minor allele	T	T	T	A	G
			AMD risk allele	C	C	C	A	G
			HDL-raising allele	T	T	C	A	G
Chen, 2010 [19]	GWA	Caucasian	Large drusen, late GA, late NV AMD, n=2157. Control, n=1150.		0.87 (0.82-0.91)	0.83 (0.76-0.89)	1.19 (1.21-1.27)	1.26 (1.11-1.43)
Neale, 2010 [20]	GWA	Caucasian	Late NV AMD, n=979. Control, n=1709.	0.82 (0.77-0.88)	0.86 (0.82-0.91)	0.77 (0.66-0.90)	1.12 (1.04-1.20)	0.85 (0.71-1.02)
Reynolds, 2010 [26]	Case-control	Caucasian	Late AMD (NV and GA), n=318. Controls, n=140.	CT: 0.6 (0.4-0.9) T: 0.4 (0.2-0.9)				
Seddon, 2010 [25]	Case-control, AREDS	Caucasian	Late AMD (NV and GA), n=545. Controls, n=275.	CT: 0.9 (0.7-1.2) TT: 0.5 (0.2-0.9)				
Fauser, 2011 [27]	Case-control	Caucasian	AMD (at least 10 small, hard drusen and pigmentary changes or at least one intermediate size drusen), n=1201. Control, n=562.	0.93 (0.78-1.09)		0.69 (0.52-0.86)	1.14(0.97-1.33)	0.98 (0.77-1.26)
Peter, 2011 [24]	Multicenter, Case-control	Caucasian	Women, Late AMD, n=146. Control, n=1269.	CT: 0.4 (0.2-0.9) TT: 0.6 (0.2-2.0)	CT: 0.8 (0.4-1.5) TT: 0.2 (0.1-0.7)	0.7 (0.4-1.3)	CA: 1.1 (0.6-2.1) AA: 1.5 (0.6-3.7)	0.6 (0.3-1.5)
Sobrin, 2011 [23]	Multicenter, Case-control	Caucasian	Late AMD (NV and GA), n=3958.	1.07 (0.94-1.23)				
Yu, 2011 [21]	Case-control, AREDS	Caucasian	Late AMD (NV and GA), n=1082. Control, n=221.	0.68 (0.53-0.87)		0.63 (0.50-0.81)	1.22 (0.97-1.55)	
Yu, 2011 [22]	Meta-analysis of case-control studies	Caucasian	Late AMD, n=2594. Control, n=4134.	0.84; p<.0001			1.15, p<.0001	
Cipriani, 2012 [30]	GWA	Caucasian	Late NV AMD or late GA, n=893. Control, n=2199.	0.91(0.80-1.03)	0.89 (0.79-0.99)		1.07 (0.95-1.19)	
Tian, 2012 [31]	Multicenter, Case-control	Han Chinese	Late AMD, n=535. Control, n=469.	OR not shown p=0.53				OR not shown p=0.94
Yu, 2012 [28]	Longitudinal study, AREDS	Caucasian	Incidence of late NV AMD (large drusen➔NV AMD)	TT: 0.64 (0.38-1.08)		1.02 (0.85-1.23)	1.27 (1.08-1.49)	
Zhang, 2013 [29]	Case-control	Han Chinese	Late NV AMD, n=157. Control, n=204.	0.75 (0.51-1.10)	0.99 (0.70-1.39)	0.65 (0.46-0.92)	1.16 (0.78-1.70)	0.86 (0.55-1.34)

Abbreviations: AREDS: Age-Related Eye Disease Study; GA: Geographic Atrophy; GWA: Genome-Wide Association; NV: Neovascular

Regarding *LIPC*, two SNPs have been studied in relation to AMD: rs493258, which was initially identified by the GWAS studies on AMD [19,32], and rs10468017, which was initially identified by a GWAS study on HDL concentrations [33]. Both SNPs, located on chromosome 15 (15q21.3), are in linkage disequilibrium (r^2^=0.42), and most studies on AMD tested only the rs10468017 SNP (Table 1). With regard to rs493258, in Caucasians, all four available studies have shown a significantly decreased risk for AMD in subjects bearing the minor (T) allele. With regard to rs10468017, in Caucasians, the risk for AMD was significantly reduced in subjects bearing the minor (T) allele in 6 studies, while 4 other studies showed no statistically significant associations. Two studies in Han Chinese showed no associations of AMD with rs493258 or rs10468017 [29,31].

As for *ABCA1*, most studies, including one study performed in Han Chinese subjects, showed a decreased risk for AMD in subjects bearing the minor (T) allele, although one study did not find any associations [28]. The *CETP* minor allele (A) was also generally associated with increased risk for AMD, although the association failed to reach statistical significance in 4 studies. Finally, results concerning *LPL* were more inconsistent: one of the original GWAS studies found a significantly increased risk for AMD [19], while the other GWAS study found a non significant decreased risk [20], consistently with three more recent case-control studies (including one in Han Chinese subjects). 

These genetic associations suggest that lipid metabolism, and in particular HDL-cholesterol, is implicated in the pathophysiology of AMD. However, the relationship between AMD and HDL cholesterol may not be straightforward. Indeed, epidemiological studies evaluating associations between plasma lipids (including HDL-cholesterol) and AMD have been inconsistent, with conflicting results [34–39]. A recent study also reported that the associations of AMD with *LIPC* and with HDL-cholesterol seem to be independent [26], thereby implying that the relationship between HDL-related genotypes and AMD may be mediated by other mechanisms. Finally, the direction of associations of AMD with HDL-related loci is not consistent: while for *LIPC*, the HDL-raising allele is associated with lower risk for AMD, while for *ABCA1* and *CETP*, they confer higher risk for AMD (Table 1). Thus, as noted by Neale et al [20], the association of *LIPC* and other HDL-related loci with AMD may not represent a causal effect of HDL cholesterol, but could rather indicate a shared underlying biological mechanism involving the cholesterol pathway. 

As explained above, the metabolism of lutein and zeaxanthin, being strongly associated both with AMD and HDL cholesterol, is a potential candidate for such common mechanism between AMD and HDL related genetic loci. However, very few data are available on the potential relationship of *LIPC* and other HDL-related genes with plasma lutein and zeaxanthin. Only one study of 129 subjects showed some associations of plasma carotenoids and vitamin E with *LIPC* and *CETP* [18]. 

Here, we assessed the associations of several genetic loci implicated in HDL metabolism with AMD and with plasma lutein and zeaxanthin concentrations in the framework of a population-based study. The main originality of the present study resides firstly, in its population-based design, which overcomes major issues in selection bias inherent to case-control studies. Secondly, we provide results for the associations of HDL-related loci with early AMD, the pre-clinical stage of AMD, while most previous studies have included only late AMD cases. Finally, we provide some data on the associations of these loci with plasma lutein and zeaxanthin, which might, at least partially, explain the association of AMD with these genetic loci.

## Subjects and Methods

### Study aims

The Alienor (Antioxydants, Lipides Essentiels, Nutrition et maladies OculaiRes) Study is a population-based study aiming at assessing the associations of age-related eye diseases (AMD, glaucoma, cataract, dry eye syndrome) with nutritional factors (in particular antioxidants, macular pigment and fatty acids), determined from estimation of dietary intakes and plasma measurements [40]. It also takes into account other major determinants of eye diseases, including gene polymorphisms, environmental and vascular factors.

This research followed the tenets of the Declaration of Helsinki. Participants gave written consent for the participation in the study. The design of the Alienor study has been approved by the Ethical Committee of Bordeaux (Comité de Protection des Personnes Sud-Ouest et Outre-Mer III) in May 2006.

### Study sample

**Figure 1:**
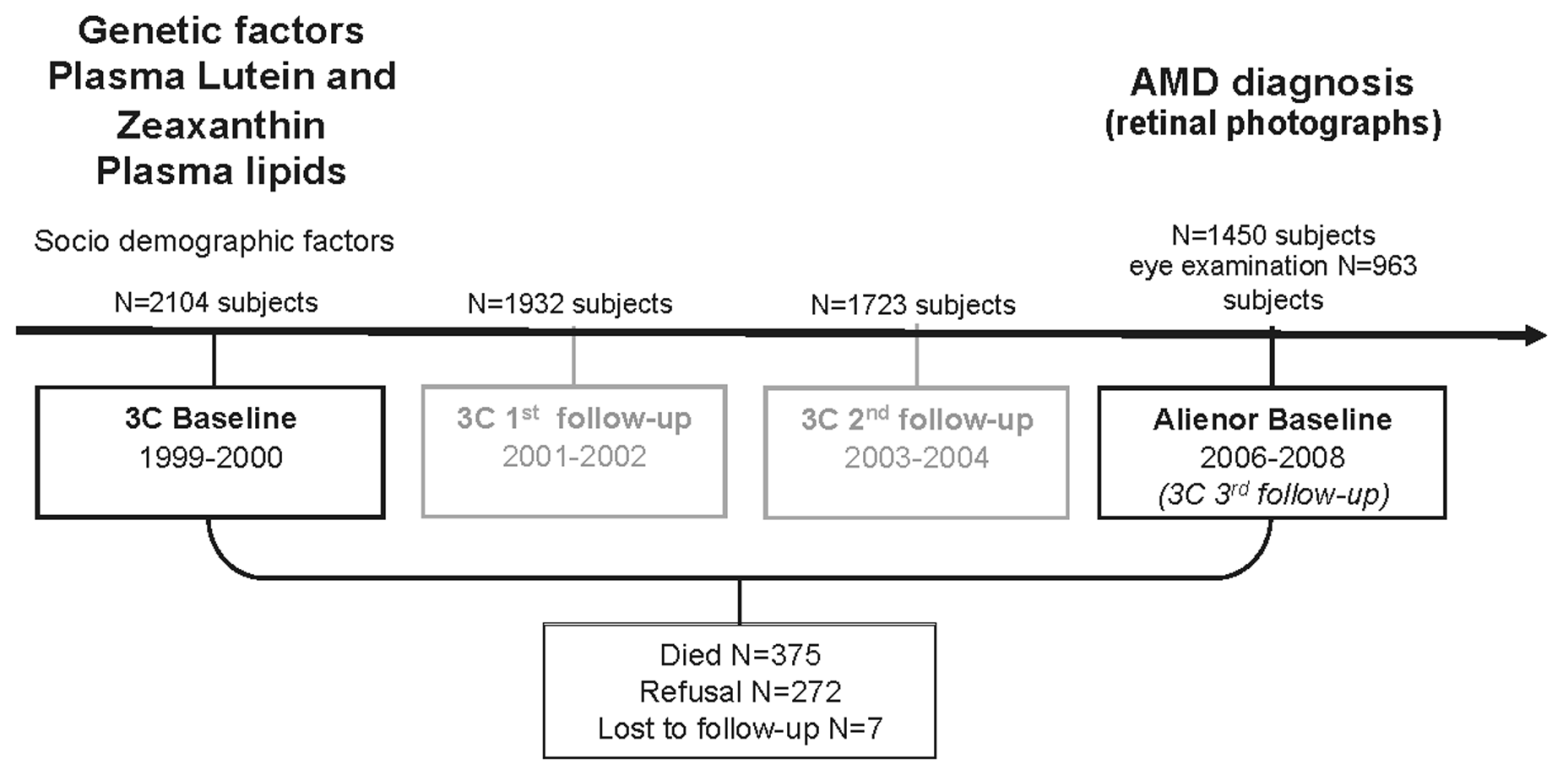
Alienor Study, data collection (1999-2008). Abbreviations: AMD: age-related macular degeneration.

Subjects of the Alienor Study were recruited from an ongoing population-based study on the vascular risk factors for dementia, the Three-City (3C) Study [41]. The 3C Study included 9294 subjects aged 65 years or more from three French Cities (Bordeaux, Dijon and Montpellier), among whom 2104 were recruited in Bordeaux. They were initially recruited in 1999-2001 and followed-up about every two years since. The Alienor Study consists of eye examinations, which are offered to all participants of the 3C cohort in Bordeaux after the third follow-up (2006-2008) (Figure **1**). Among the 1450 participants re-examined between October 2006 and May 2008, 963 (66.4%) participated in the Alienor Study’s baseline eye examination. 

### Eye examination

The eye examination took place in the Department of Ophthalmology of the University Hospital of Bordeaux at Alienor baseline (2006-2008). It included a recording of ophthalmological history, measures of visual acuity, refraction, two 45° non mydriatic colour retinal photographs (one centered on the macula, the other centered on the optic disc), measures of intraocular pressure and central corneal thickness and break-up time test. 

Retinal photographs were performed using a non mydriatic retinograph (TRC NW6S, Topcon, Japan) and were interpreted in duplicate by two specially trained technicians. Inconsistencies between the two interpretations were adjudicated by a senior grader. Finally, all cases of late AMD were reviewed and confirmed by retina specialists (JFK, MND and MBR).

None of the persons implicated in interpretation of retinal photographs had access to plasma measurements, genetic determinations or information on risk factors.

### Classification of AMD

Retinal photographs were interpreted according to the international classification [42] and to a modification of the grading scheme used in the Multi-Ethnic Study of Atherosclerosis for drusen size, location and area [43]. Late AMD was defined by the presence of neovascular AMD or geographic atrophy within the grid (3000 microns from the *Foveola*). Neovascular AMD included serous or hemorrhagic detachment of the retinal pigment epithelium (RPE) or sensory retina, sub-retinal or sub-RPE hemorrhages and fibrous scar tissue. Geographic atrophy was defined as a discrete area of retinal depigmentation, 175 microns in diameter or larger, characterized by a sharp border and the presence of visible choroidal vessels. Five cases of late AMD had no gradable photographs and were classified by using ophthalmological history of AMD and AMD therapy (in particular antiangiogenic agents and photodynamic therapy), and confirmed by their treating ophthalmologist.

Early AMD was defined by the presence of soft distinct drusen and/or soft indistinct drusen and/or reticular drusen and/or pigmentary abnormalities. Soft distinct and indistinct drusen were larger than 125 microns in diameter and with uniform density and sharp edges or decreasing density from the center outwards and fuzzy edges, respectively. Pigmentary abnormalities were defined as areas of hyperpigmentation and/or hypopigmentation (without visibility of choroidal vessels). 

### Genotyping

Genotyping was performed on DNA extracted from leukocytes at baseline (1999-2001) and kept frozen at -80°C. Centralized facilities for genotyping are provided by the Lille Genopôle. Genotyping of single nucleotide polymorphisms (SNPs) rs1061170 (Complement Factor H, *CFH*, Y402H) were determined using TaqMan assays (Applied Biosystems, Inc., [ABI], Foster City, CA), as described by the suppliers. Furthermore, a genome wide scan was performed at the Lille Genopôle [44]. For the present study, the following genotypes were extracted from the GWAS data: rs10490924 (*ARMS2/HTRA1*), rs493258 and rs10468017 (*LIPC*), rs3764261 (*CETP*), rs12678919 (*LPL*) and rs1883025 (*ABCA1*). Genotypes data for rs10468017 (*LIPC*), rs3764261 (*CETP*) and rs12678919 (*LPL*) were available. For the others SNPs namely rs10490924 (*ARMS2/HTRA1*), rs493258 (*LIPC*), rs9621532 and rs1883025 (*ABCA1*), genotypes were imputed using Markov Chain based haplotyper (MACH v1.0.16a) [45,46] software and the 1000 Genomes Project data [47] (r^2^=0.96; r^2^=0.94; r^2^=0.99 and r^2^=0.96, for rs10490924, rs493258, rs9621532 and rs1883025, respectively).

### Plasma measurements

Plasma measurements were determined from fasting blood samples collected at the 3C baseline visit (1999-2001) into heparinized evacuated tubes and centrifuged at 1000 g for 10 min. Plasma lipids (HDL and LDL-cholesterol, triglycerides) were measured at the Biochemistry Laboratory of the University Hospital of Dijon from baseline fasting blood samples.

Plasma lutein and zeaxanthin measurements were performed at DSM Nutritional Products (Kaiseraugst, Switzerland). Their concentrations were determined by normal-phase HPLC, using dedicated analytical methods [48]. Plasma samples were analysed for zeaxanthin (sum of all-E and Z-isomers) and lutein (sum of all-E and Z-isomers). The xanthophylls were extracted from plasma (100 mL) with a 20% mixture of n-hexane and chloroform (1100 mL) after dilution with water (100 mL) and proteins precipitation with ethanol (200 mL). After centrifugation, an aliquot (800 mL) of the clear supernatant fluid was dried under nitrogen at room temperature. The dried residue was quantitatively redissolved in the mobile phase (200 mL n-hexane and acetone; 19%, by vol). The resulting solution was injected (100 mL) into a normal-phase HPLC system equipped with an autosampler (15°C), a column oven (40°C), an HPLC pump, and an ultraviolet-visible detector. Data were analysed with a data acquisition system (Atlas: Thermo Labsystems). The separation was done on a polar column (Lichrosorb, Si60, 5 mm, 250 * 4 mm; Stagroma, Reinach, Switzerland) with a mixture of n-hexane and acetone (19%, by vol) at a flow rate of 1 mL/min. Xanthophylls were detected at a wavelength of 452 nm.  To assess the daily and long-term laboratory performance of the HPLC plasma analytics, dedicated control plasma was used. None of the people involved in plasma carotenoid determination had any access to ocular clinical findings or genetic data at any time of the study. 

### Covariates

Socio-demographic and lifestyle data were collected through face-to-face standardized interview in 1999-2001. They included age, gender and smoking (never smoker, <20 pack-years (PY) and ≥20 PY, where number of PY = packs (20 cigarettes) smoked per day X years of smoking).

### Statistical analyses

For comparison between subjects included and not included in analyses, Student test was used for quantitative variables and Chi^2^ test for qualitative variables. 

Associations of AMD with all genetic polymorphisms were estimated using logistic Generalized Estimating Equations (GEE) models [49], which allow taking into account the data from both eyes and their intra-individual correlations. We used information for eyes instead of subjects in order to increase statistical power for AMD analyses. For each genetic polymorphism, odds-ratios adjusted for age and gender were estimated using early AMD, and late AMD as the dependent variable, and the SNP genotype variable, age, gender as the independent variables. Odds-ratios adjusted for potential confounders were obtained by adding these confounders as independent variables to the models. Potential confounders retained in the final multivariate models were factors strongly associated with AMD in our cohort (smoking [50], *CFH* [50] and *ARMS2* [51] polymorphisms). Among 963 subjects, 76 (7.9%) had ungradable photographs in both eyes, 53 (5.5%) refused the blood sampling and 8 (0.8%) had missing data for photographs and blood sampling. Among the 826 remaining subjects, available data for the different SNPs were somewhat variable, leaving between 715 and 737 subjects for each analysis.

Associations of SNPs genotype with plasma lipids (triglycerides, HDL and LDL-cholesterol) and lutein and zeaxanthin were performed using analysis of variance adjusted for age, gender and lipid lowering medication. Correlations of plasma lutein and zeaxanthin with plasma lipids were performed using Pearson correlations.

Associations between plasma lutein and zeaxanthin with *LIPC* and *LPL* SNPs were evaluated with linear regression models. Plasma lutein and plasma zeaxanthin were used as the dependent variable and the *LIPC* and *LPL g*enotypes, age, gender, HDL-cholesterol, LDL-cholesterol, triglycerides and lipid lowering medication as the independent variables. Among the 963 participants, 108 (11.2%) participants had missing data for plasma lutein and zeaxanthin: 61 (6.3 %) refused the blood sampling and 47 (4.9%) had unavailable data for plasma lutein and zeaxanthin. Among the 855 remaining subjects, the LIPC rs493258 genotype was available in 761 subjects, the LIPC rs10468017 in 748 and the LPL rs12678919 genotype in 738 subjects.

For AMD variables, eyes with no AMD were the reference. For SNPs genotype, the most frequent polymorphism was used as a reference. Quantitative variables (age, plasma lutein and zeaxanthin, plasma HDL-cholesterol, LDL-cholesterol and triglycerides) were handled as continuous variables in all analyses. 

All statistical analyses were performed using SAS version 9.2 (SAS Institute Inc, Cary, NC; procedure GENMOD for the GEE analysis).

## Results

Table 2 showed baseline characteristics of Alienor participants. Mean of age was 80.2 years (± 4.4), 62.0 % of the sample were women and 32.8 % declared lipid lowering medication use. 64.6 % had never smoked, 18.2 % had smoked less than 20 pack-years and 17.2 % more than 20 pack-years. Mean of BMI was 26.4 kg/m^2^ (± 3.9). Means of plasma lutein and zeaxanthin were 166.6 (± 87.5) and 40.8 (± 30.2) µg/L.

**Table 2:** Baseline characteristics of Alienor sample.

	Alienor sample
Socio-demographic and lifestyle characteristics	
Age (yrs) n	963
Mean (sd)	80.2 (4.4)
Gender (wowen) n	963
n (%)	597 (62.0)
Smoking n (%)	951
Never	614 (64.6)
< 20 pack-years	173 (18.2)
≥ 20 pack-years	164 (17.2)
Lipid lowering medication (yes) n	963
n (%)	316 (32.8)
Body Mass Index (kg/m^2^) n	955
Mean (sd)	26.4 (3.9)
AMD characteristics n	
n (%)	879
No AMD	580 (66.0)
Early AMD	250 (28.4)
Late AMD	49 (5.6)
Plasma characteristics, mean (sd)	
Lutein (µg/L), n=855	166.6 (87.5)
Zeaxanthin (µg/L), n=855	40.8 (30.2)
Lutein+zeaxanthin (µg/L) n=855	207.3 (108.6)
HDL-cholesterol (mmol/L), n=901	1.59±0.39
LDL-cholesterol (mmol/L), n=901	3.63 (0.85)
Total cholesterol (mmol/L), n=901	5.78±0.98
Triglycerides (mmol/L), n=901	1.23±0.60
Genetic polymorphism, n (%)	
LIPC rs49325	N=806
CC	237 (29.4)
CT	389 (48.3)
TT	180 (22.3)
LIPC rs10468017	N=792
CC	415 (52.4)
CT	315 (39.8)
TT	62 (7.8)
APOE	N=889
At least 1 Allele E2	120 (13.5)
At least 1 Allele E4	159 (17.9)
ABCA1 rs1883025	N=806
CC	422 (52.4)
CT	320 (39.7)
TT	64 (7.9)
LPL rs12678919	N=782
AA	581 (74.3)
AG	186 (23.8)
GG	15 (1.9)
CETP rs3764261	N=788
CC	415 (52.7)
AC	310 (39.3)
AA	63 (8.0)
CFH	N=878
CC	399 (45.4)
CT	378 (43.1)
TT	101 (11.5)
ARMS2	N=806
GG	524 (65.0)
GT	254 (31.5)
TT	28 (3.5)

Alienor Study Bordeaux, France (2006-2008).

As shown in Table 3, after adjustment for age and gender, the TT genotype of *LIPC* rs493258 SNP was associated with a decreased risk for early and late AMD (model 1). These associations remained significant after further adjustment for smoking, *CFH* and *ARMS2* genotypes and lipid lowering medication (model 2). The CT genotype of *LIPC* rs10468017 SNP was significantly associated with a decreased risk for early AMD in model 1. However, statistical significance was not maintained after full adjustment. The associations of the CT genotype with late AMD and of the TT genotype with early and late AMD were in the same direction, but far from significant. We found an inverse association between the AG genotype of *LPL* polymorphism and early AMD in model 1, but these associations were of borderline significance (p=0.05) after further adjustment for smoking, *CFH*, *ARMS2* and lipid lowering medication. No associations were found between *ABCA1* and *CETP* polymorphisms and early or late AMD.

**Table 3:** Associations between AMD and genetic polymorphisms of lipids metabolism genes.

	Model 1: OR adjusted for age and gender			Model 2: OR adjusted for age, gender, smoking, CFH and ARMS2 polymorphism, lipid lowering medication		
	No AMD	Early AMD	Late AMD	No AMD	Early AMD	Late AMD
*LIPC* rs493258 (n)	1062	292	70	1031	281	66
CC (n=413)		1.0 (ref)	1.0 (ref)		1.0 (ref)	1.0 (ref)
CT (n=687), OR		0.83	0.82		0.88	0.85
(95 % CI)		(0.58-1.18)	(0.42-1.62)		(0.61-1.28)	(0.41-1.77)
p-value		0.30	0.57		0.51	0.67
TT (n=324), OR		0.60	0.28		0.64	0.25
(95 % CI)		(0.39-0.92)	(0.09-0.83)		(0.41-0.99)	(0.08-0.84)
p-value		0.02	0.02		0.046	0.03
*LIPC* rs10468017 (n)	1043	285	70	996	272	66
CC (n=717)		1.0 (ref)	1.0 (ref)		1.0 (ref)	1.0 (ref)
CT (n=564), OR		0.70	0.64		0.72	0.58
(95 % CI)		(0.50-0.98)	(0.32-1.25)		(0.51-1.02)	(0.28-1.16)
p-value		0.04	0.19		0.07	0.12
TT (n=117), OR		0.76	0.40		0.73	0.21
(95 % CI)		(0.44-1.31)	(0.09-1.79)		(0.41-1.29)	(0.03-1.58)
p-value		0.33	0.23		0.27	0.13
*LPL* rs12678919 (n)	1025	287	68	996	276	64
AA (n=1023)		1.0 (ref)	1.0 (ref)		1.0 (ref)	1.0 (ref)
AG (n=331), OR		0.63	0.81		0.61	0.93
(95 % CI)		(0.42-0.93)	(0.37-1.80)		(0.45-1.00)	(0.41-2.13)
p-value		0.02	0.61		0.05	0.87
GG (n=36), OR		1.43	2.44		1.41	3.06
(95 % CI)		(0.48-4.23)	(0.59-10.09)		(0.47-4.18)	(0.75-12.53)
p-value		0.52	0.22		0.54	0.12
*ABCA1* rs1883025 (n)	1062	292	70	1031	281	66
CC (n=755)		1.0 (ref)	1.0 (ref)		1.0 (ref)	1.0 (ref)
CT (n=560), OR		0.97	0.80		1.01	0.62
(95 % CI)		(0.70-1.34)	(0.41-1.55)		(0.73-1.42)	(0.30-1.28)
p-value		0.84	0.51		0.93	0.19
TT (n=109), OR		1.04	0.48		1.18	0.41
(95 % CI)		(0.60-1.81)	(0.10-2.30)		(0.65-2.12)	(0.09-1.85)
p-value		0.89	0.36		0.59	0.25
*CETP* rs3764261 (n)	1035	287	68	1004	276	64
CC (n=730)		1.0 (ref)	1.0 (ref)		1.0 (ref)	1.0 (ref)
AC (n=558), OR		1.17	1.04		1.24	1.29
(95 % CI)		(0.85-1.61)	(0.53-2.03)		(0.89-1.73)	(0.63-2.66)
p-value		0.32	0.91		0.20	0.49
AA (n=102), OR		0.59	1.38		0.54	1.52
(95 % CI)		(0.29-1.20)	(0.42-4.54)		(0.25-1.15)	(0.44-5.22)
p-value		0.14	0.60		0.11	0.51

Alienor Study (Bordeaux, France, 2006-2008)

**Figure 2:**
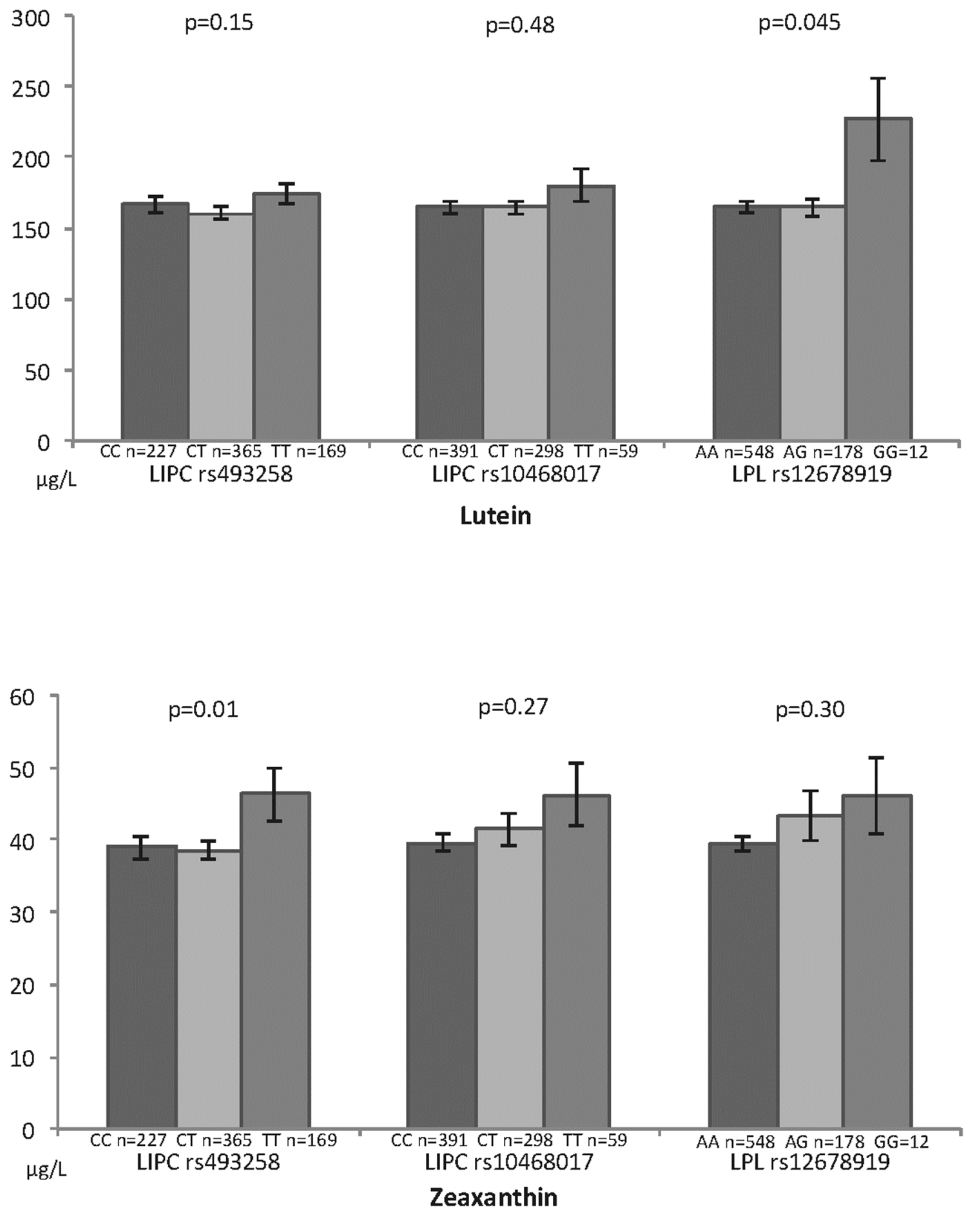
Associations between plasma lutein, zeaxanthin and genetic polymorphisms of lipids metabolism. Alienor Study, Bordeaux, France. P Anova adjusted for age and gender and lipid lowering medication.

P value for generalized estimating equation model. Abbreviations: AMD: age-macular degeneration;

Figure 2 displays the associations of plasma lutein and zeaxanthin with *LIPC* rs493258 and rs10468017 and *LPL* rs12678919 SNPs. Plasma lutein was increased in the GG genotype of *LPL* (p=0.046), while zeaxanthin was associated with *LIPC* rs493258 (p=0.01) but not with *LIPC* rs10468017 or *LPL* genotypes. The TT genotype of *LIPC* rs493258 had the highest mean of plasma zeaxanthin.

**Figure 3:**
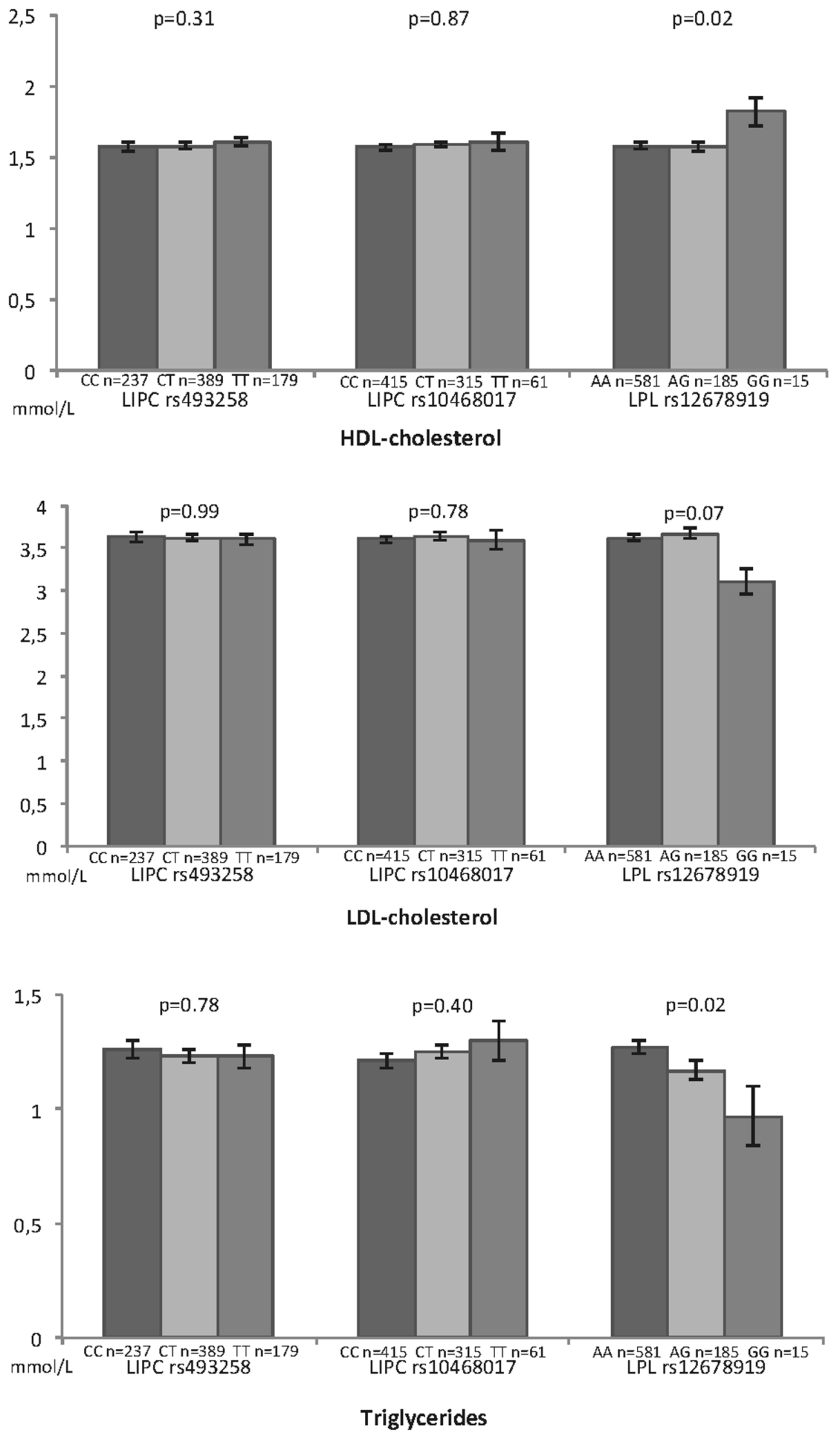
Associations between plasma lipids and genetic polymorphisms of lipids metabolism. Alienor Study Bordeaux, France. P Anova adjusted for age, gender and lipid lowering medication.

Figure 3 depicts the associations of plasma lipids with *LIPC* rs493258 and rs10468017 and *LPL* rs12678919 SNPs. We found no differences in mean for any plasma lipids with *LIPC* rs493258 or rs10468017 polymorphisms. With regard to the *LPL* polymorphism, LDL-cholesterol and triglycerides were significantly lower for subjects with GG genotype (respectively p=0.05 and p=0.03), whereas HDL-cholesterol was significantly higher for GG carriers (p=0.02).

As shown in Table 4, plasma lutein and zeaxanthin significantly correlated with plasma cholesterol, and in particular HDL-cholesterol, while they correlated negatively with plasma triglycerides.

**Table 4:** Correlations of plasma lutein and zeaxanthin with plasma lipids.

	Zeaxanthin	Lutein + zeaxanthin	HDL-cholesterol	LDL-cholesterol	Total cholesterol	Triglycerides
Lutein	0.61 (<0.0001)^a^	0.98 (<0.0001)	0.30 (<0.0001)	0.16 (<0.0001)	0.21(<0.0001)	-0.16 (<0.0001)
Zeaxanthin		0.77 (<0.0001)	0.20 (<0.0001)	0.18 (<0.0001)	0.21 (<0.0001)	-0.12 (0.0007)
Lutein + zeaxanthin			0.30 (<0.0001)	0.18 (<0.0001)	0.23 (<0.0001)	-0.16 (<0.0001)

^a^ Pearson Coefficient correlation (P value)

Pearson correlation. Alienor Study (Bordeaux, France, 2006-2008), n=855.

As shown in Table 5, associations of *LIPC* rs493258 TT genotype with plasma zeaxanthin and *LPL* rs1267819 with plasma lutein were maintained after further adjustment of plasma lipids and lipid lowering medication.

**Table 5:** Associations between plasma lutein and zeaxanthin with *LIPC* and *LPL* polymorphisms.

	Plasma Lutein (µg/l)				Plasma Zeaxanthin (µg/l)			
LIPC rs493258, n=759	Mean (SD)	Difference^a^	CI 95%	P	Mean (SD)	Difference^a^	CI 95%	P
CC reference, n=227	166.9 (92.5)	-	-	-	38.9 (24.5)	-	-	-
CT (/CC), n=364	161.2 (84.1)	-7.82	-21.61 - 5.96	0.27	38.5 (22.9)	-0.82	-5.77 - 4.13	0.75
TT (/CC), n=168	173.2 (89.4)	3.82	-12.76 - 20.39	0.65	46.3 (48.2)	6.78	0.83 - 12.74	0.03
LIPC rs10468017, n=746								
CC reference, n=391	165.1 (89.6)	-	-	-	39.6 (24.8)	-	-	-
CT (/CC), n=297	165.2 (85.0)	-1.53	-13.96 - 10.91	0.81	41.5 (37.7)	1.38	-3.18 - 5.93	0.55
TT (/CC), n=58	178.7 (88.2)	13.09	-9.65 - 35.83	0.26	46.2 (33.0)	6.87	-1.46 - 15.19	0.11
LPL rs1267819, n=736								
AA reference, n=547	164.8 (87.0)	-	-	-	39.5 (24.8)	-	-	-
AG (/AA), n=177	163.9 (82.6)	-1.49	-15.41 - 12.43	0.83	43.2 (45.3)	3.13	-1.98 - 8.25	0.23
GG (/AA), n=12	226.8 (100.6)	47.76	0.48 - 95.03	0.047	46.1 (18.3)	4.64	-12.72 - 22.01	0.60

Adjusted linear regressions. Alienor Study (Bordeaux, France, 2006-2008).

^a^ Adjusted for age, gender, HDL-cholesterol, LDL-cholesterol, plasma triglycerides and lipid lowering medication using multivariate linear regression.

## Discussion

This study confirms the association of AMD with *LIPC*, and suggests that an association of *LIPC* with the metabolism of lutein and zeaxanthin could, at least partly, mediate it. With regard to *LIPC* rs493258, our study was consistent with five previous studies in Caucasians, showing a decreased risk for late AMD in subjects bearing the T allele. Consistently with one previous study [24], association with *LIPC* rs493258 was significant for subjects with the TT genotype but not for those with the CT genotype, suggesting a recessive mode of effect. Moreover, we also identified a significantly reduced risk for early AMD, also on a recessive mode. 

Although our results for *LIPC* rs10468017 did not reach statistical significance, they were consistent with other studies, showing a decreased risk for AMD in subjects bearing the T allele [21,22,24–26]. Three other studies in Caucasians did not show significant associations of *LIPC* rs10468017 with AMD [23,27,28]. Although the rs10468017 and rs493258 are linkage disequilibrium (LD) (r^2^=0.42), *LIPC* functional variant(s), tagged by rs10468017 and rs493258 markers have not been identified yet, and therefore it is possible that rs493258 may be in LD with the functional variant, whereas rs10468017 SNP, which is further in the promoter region, may not be. Regarding the effect of *LIPC* variants on HDL-cholesterol level, tedious investigations have highlighted functional variants such as rs1800588 (-514C>T) and variant rs493258 is in LD with rs1800588 but rs10468017 is not. 

In our study, we found a significant association between plasma zeaxanthin concentration and *LIPC* rs493258 variant, but not with *LIPC* rs10468017 SNP, after adjustment for potential confounders, including plasma lipids. Lutein and zeaxanthin are fat-soluble micronutrients carried by plasma lipoprotein [52], mainly by HDL [15]. As expected, in the present study, plasma lutein and zeaxanthin correlated with plasma cholesterol, and in particular HDL-cholesterol. It is well established that lutein and zeaxanthin absorption is not passive and some genes like *SCARB1, ABCG5, BCMO1* and *CD36* are implicated in their transport across cellular membranes [16–18]. In a French study, the *LIPC* rs1800588 variant was associated with plasma concentrations of some carotenoids (γ-tocopherol, α-carotene and β-carotene) but not with xanthophylls [18]. By contrast, we report an association of *LIPC* rs493258 variant with plasma zeaxanthin, but not with plasma lutein or other carotenoids (data not shown). Finally, a recent study showed a variation of macular pigment density (formed by lutein and zeaxanthin in the retina) according to another LIPC polymorphism (rs6078) [53]. Different variants of the hepatic lipase gene could therefore interfere in different ways with the metabolism of specific carotenoids.

Regarding the *LPL* gene, one GWAS [20] and three case-control studies [24,27,29] found a non-significant decreased risk for AMD in G allele carriers of the rs1267819 SNP. By contrast, one study [19] found a significant increased risk for AMD in G carriers. Our results for late AMD are consistent with this last study: we showed an increased risk for late AMD for the GG genotype, which did not reach statistical significance, probably because of small sample size for GG. On the contrary, we found a borderline decreased risk for early AMD in AG carriers.

With regard to *CETP* and *ABCA1* genes, two initial GWAS [19,20] reported significant associations with AMD. Results from the present study were in the same direction (decreased risk for *ABCA1* rs1883025 T carriers and increased risk for *CETP* rs3764261 A carriers), but did not reach statistical significance. The relatively small sample size may have impeded us to detect these associations, the minor alleles being less frequent for *CETP* and *ABCA1* than for *LIPC* rs493258.

Alienor participants seem to have higher levels of plasma lutein and zeaxanthin than AREDS2 participants [54]: 207.3 (± 108.6) versus 179.0 95 % CI (171.0-188.0) µg/L, possibly resulting from differences in dietary habits.

Strengths of our study include the population-based design, with photographic assessment of AMD in all subjects. Previous studies of associations of AMD with HDL-related loci were all case-control, hospital-based studies, where selection bias is a major issue, in particular for controls. Moreover, in other studies, definition of AMD cases was not always clear and control choice maybe questionable. In the present study, we used the international classification of AMD, based on retinal photographs, and distinguished early from late AMD. 

A potential limitation to our results is the questionable representativeness of the sample. First, about two thirds of the participants in The 3C Study accepted the eye examination. However, subjects included in the Alienor study were not different from those who did not participate for most parameters of interest in our study [40]. Moreover, the prevalence of AMD in our study was similar to that observed in the same age group in other studies performed in Europe [2,55] and other industrialized countries [3] and the distribution of genetic polymorphisms was similar to that observed in other studies in Caucasians [21–27]. We also observed expected associations of AMD with major risk factors (smoking, *CFH* and *ARMS2*) in this sample [50,51]. 

The relatively small number of late AMD cases (n=70 eyes) limited statistical power for analysis of subcategories (atrophic/neovascular). In addition, associations of *LIPC* rs10468017, *CETP* rs3764261 and *ABCA1* rs1883025 with early and late AMD did not reach statistical significance in our sample, although the estimated odds-ratios were in the expected range. Finally, a potential limitation of our study is the high number of comparisons performed. Therefore, we cannot exclude that some of the observed associations were due to chance finding, although our findings are generally consistent with previous studies in this field.

In conclusion, the present population-based study confirms the association of *LIPC* polymorphism with AMD and suggests a potential association between *LIPC* and plasma zeaxanthin concentrations. We also detected associations of *LPL* with AMD and plasma lutein. These findings suggest that *LIPC* and *LPL* may be implicated both in AMD pathogenesis and lutein and zeaxanthin transport. Further study of the *LIPC* locus may contribute to our understanding and points another pathway in the pathogenesis of AMD. These findings open up new opportunities in the research for prevention and treatment of AMD. Further studies, in particular large prospective population-based studies, will be needed to confirm these findings.

## References

[1] Resnikoff S, Pascolini D, Etya'ale D, Kocur I, Pararajasegaram R et al. (2004) Global data on visual impairment in the year 2002. Bull World Health Organ 82: 844-851. PubMed: 15640920. 15640920 PMC2623053

[2] Augood CA, Vingerling JR, de Jong PT, Chakravarthy U, Seland J et al. (2006) Prevalence of age-related maculopathy in older Europeans: the European Eye Study (EUREYE). Arch Ophthalmol 124: 529-535. doi:10.1001/archopht.124.4.529. PubMed: 16606879. 16606879

[3] Friedman DS, O'Colmain BJ, Muñoz B, Tomany SC, McCarty C et al. (2004) Prevalence of age-related macular degeneration in the United States. Arch Ophthalmol 122: 564-572. doi:10.1001/archopht.122.4.564. PubMed: 15078675. 15078675

[4] Lim LS, Mitchell P, Seddon JM, Holz FG, Wong TY (2012) Age-related macular degeneration. Lancet 379: 1728-1738. doi:10.1016/S0140-6736(12)60282-7. PubMed: 22559899. 22559899

[5] Chong EW, Kreis AJ, Wong TY, Simpson JA, Guymer RH (2008) Dietary omega-3 fatty acid and fish intake in the primary prevention of age-related macular degeneration: a systematic review and meta-analysis. Arch Ophthalmol 126: 826-833. doi:10.1001/archopht.126.6.826. PubMed: 18541848. 18541848

[6] Thornton J, Edwards R, Mitchell P, Harrison RA, Buchan I et al. (2005) Smoking and age-related macular degeneration: a review of association. Eye 19: 935-944. doi:10.1038/sj.eye.6701978. PubMed: 16151432. 16151432

[7] Chan D (1998) Cigarette smoking and age-related macular degeneration. Optom Vis Sci 75: 476-484. doi:10.1097/00006324-199807000-00015. PubMed: 9703035. 9703035

[8] van Leeuwen R, Boekhoorn S, Vingerling JR, Witteman JC, Klaver CC et al. (2005) Dietary intake of antioxidants and risk of age-related macular degeneration. JAMA 294: 3101-3107. doi:10.1001/jama.294.24.3101. PubMed: 16380590. 16380590

[9] Kijlstra A, Tian Y, Kelly ER, Berendschot TT (2012) Lutein: more than just a filter for blue light. Prog Retin Eye Res 31: 303-315. doi:10.1016/j.preteyeres.2012.03.002. PubMed: 22465791. 22465791

[10] Whitehead AJ, Mares JA, Danis RP (2006) Macular pigment: a review of current knowledge. Arch Ophthalmol 124: 1038-1045. doi:10.1001/archopht.124.7.1038. PubMed: 16832030. 16832030

[11] Delcourt C, Carrière I, Delage M, Barberger-Gateau P, Schalch W (2006) Plasma lutein and zeaxanthin and other carotenoids as modifiable risk factors for age-related maculopathy and cataract: the POLA Study. Invest Ophthalmol Vis Sci 47: 2329-2335. doi:10.1167/iovs.05-1235. PubMed: 16723441. 16723441

[12] (1993) Antioxidant status and neovascular age-related macular degeneration Eye Disease Case -control study group. Arch Ophthalmol 111: 104-109

[13] Gale CR, Hall NF, Phillips DI, Martyn CN (2003) Lutein and zeaxanthin status and risk of age-related macular degeneration. Invest Ophthalmol Vis Sci 44: 2461-2465. doi:10.1167/iovs.02-0929. PubMed: 12766044. 12766044

[14] Ma L, Dou HL, Wu YQ, Huang YM, Huang YB et al. (2012) Lutein and zeaxanthin intake and the risk of age-related macular degeneration: a systematic review and meta-analysis. Br J Nutr 107: 350-359. doi:10.1017/S0007114511004260. PubMed: 21899805. 21899805

[15] Clevidence BA, Bieri JG (1993) Association of carotenoids with human plasma lipoproteins. Methods Enzymol 214: 33-46. doi:10.1016/0076-6879(93)14051-J. PubMed: 8469147. 8469147

[16] Borel P (2012) Genetic variations involved in interindividual variability in carotenoid status. Mol Nutr Food Res 56: 228-240. doi:10.1002/mnfr.201100322. PubMed: 21957063. 21957063

[17] Borel P, de Edelenyi FS, Vincent-Baudry S, Malezet-Desmoulin C, Margotat A et al. (2011) Genetic variants in BCMO1 and CD36 are associated with plasma lutein concentrations and macular pigment optical density in humans. Ann Med 43: 47-59. doi:10.3109/07853890.2011.586359. PubMed: 21091228. 21091228

[18] Borel P, Moussa M, Reboul E, Lyan B, Defoort C et al. (2009) Human fasting plasma concentrations of vitamin E and carotenoids, and their association with genetic variants in apo C-III, cholesteryl ester transfer protein, hepatic lipase, intestinal fatty acid binding protein and microsomal triacylglycerol transfer protein. Br J Nutr 101: 680-687. doi:10.1017/S0007114508030754. PubMed: 18662427. 18662427

[19] Chen W, Stambolian D, Edwards AO, Branham KE, Othman M et al. (2010) Genetic variants near TIMP3 and high-density lipoprotein-associated loci influence susceptibility to age-related macular degeneration. Proc Natl Acad Sci U S A 107: 7401-7406. doi:10.1073/pnas.0912702107. PubMed: 20385819. 20385819 PMC2867722

[20] Neale BM, Fagerness J, Reynolds R, Sobrin L, Parker M et al. (2010) Genome-wide association study of advanced age-related macular degeneration identifies a role of the hepatic lipase gene (LIPC). Proc Natl Acad Sci U S A 107: 7395-7400. doi:10.1073/pnas.0912019107. PubMed: 20385826. 20385826 PMC2867697

[21] Yu Y, Reynolds R, Fagerness J, Rosner B, Daly MJ et al. (2011) Association of variants in the LIPC and ABCA1 genes with intermediate and large drusen and advanced age-related macular degeneration. Invest Ophthalmol Vis Sci 52: 4663-4670. doi:10.1167/iovs.10-7070. PubMed: 21447678. 21447678 PMC3175969

[22] Yu Y, Bhangale TR, Fagerness J, Ripke S, Thorleifsson G et al. (2011) Common variants near FRK/COL10A1 and VEGFA are associated with advanced age-related macular degeneration. Hum Mol Genet 20: 3699-3709. doi:10.1093/hmg/ddr270. PubMed: 21665990. 21665990 PMC3159552

[23] Sobrin L, Reynolds R, Yu Y, Fagerness J, Leveziel N et al. (2011) ARMS2/HTRA1 locus can confer differential susceptibility to the advanced subtypes of age-related macular degeneration. Am J Ophthalmol 151: 345-352 e343 doi:10.1016/j.ajo.2010.08.015. PubMed: 21122828. 21122828 PMC3763907

[24] Peter I, Huggins GS, Ordovas JM, Haan M, Seddon JM (2011) Evaluation of new and established age-related macular degeneration susceptibility genes in the Women's Health Initiative Sight Exam (WHI-SE) Study. Am J Ophthalmol 152: 1005-1013 e1001 doi:10.1016/j.ajo.2011.05.016. PubMed: 21906714. 21906714 PMC4446967

[25] Seddon JM, Reynolds R, Rosner B (2010) Associations of smoking, body mass index, dietary lutein, and the LIPC gene variant rs10468017 with advanced age-related macular degeneration. Mol Vis 16: 2412-2424. PubMed: 21139980. 21139980 PMC2994762

[26] Reynolds R, Rosner B, Seddon JM (2010) Serum lipid biomarkers and hepatic lipase gene associations with age-related macular degeneration. Ophthalmology 117: 1989-1995. doi:10.1016/j.ophtha.2010.07.009. PubMed: 20888482. 20888482 PMC3081670

[27] Fauser S, Smailhodzic D, Caramoy A, van de Ven JP, Kirchhof B et al. (2011) Evaluation of serum lipid concentrations and genetic variants at high-density lipoprotein metabolism loci and TIMP3 in age-related macular degeneration. Invest Ophthalmol Vis Sci 52: 5525-5528. doi:10.1167/iovs.10-6827. PubMed: 21613373. 21613373

[28] Yu Y, Reynolds R, Rosner B, Daly MJ, Seddon JM (2012) Prospective assessment of genetic effects on progression to different stages of age-related macular degeneration using multistate Markov models. Invest Ophthalmol Vis Sci 53: 1548-1556. doi:10.1167/iovs.11-8657. PubMed: 22247473. 22247473 PMC3339916

[29] Zhang X, Li M, Wen F, Zuo C, Chen H et al. (2013) Different impact of high-density lipoprotein-related genetic variants on polypoidal choroidal vasculopathy and neovascular age-related macular degeneration in a Chinese Han population. Exp Eye Res 108: 16-22. doi:10.1016/j.exer.2012.12.005. PubMed: 23274582. 23274582

[30] Cipriani V, Leung HT, Plagnol V, Bunce C, Khan JC et al. (2012) Genome-wide association study of age-related macular degeneration identifies associated variants in the TNXB-FKBPL-NOTCH4 region of chromosome 6p21.3. Hum Mol Genet 21: 4138-4150. doi:10.1093/hmg/dds225. PubMed: 22694956. 22694956 PMC3428154

[31] Tian J, Yu W, Qin X, Fang K, Chen Q et al. (2012) Association of genetic polymorphisms and age-related macular degeneration in Chinese population. Invest Ophthalmol Vis Sci 53: 4262-4269. doi:10.1167/iovs.11-8542. PubMed: 22618592. 22618592

[32] Maller J, George S, Purcell S, Fagerness J, Altshuler D et al. (2006) Common variation in three genes, including a noncoding variant in CFH, strongly influences risk of age-related macular degeneration. Nat Genet 38: 1055-1059. doi:10.1038/ng1873. PubMed: 16936732. 16936732

[33] Kathiresan S, Willer CJ, Peloso GM, Demissie S, Musunuru K et al. (2009) Common variants at 30 loci contribute to polygenic dyslipidemia. Nat Genet 41: 56-65. doi:10.1038/ng.291. PubMed: 19060906. 19060906 PMC2881676

[34] van Leeuwen R, Tomany SC, Wang JJ, Klein R, Mitchell P et al. (2004) Is medication use associated with the incidence of early age-related maculopathy? Pooled findings from 3 continents. Ophthalmology 111: 1169-1175. doi:10.1016/j.ophtha.2003.10.024. PubMed: 15177967. 15177967

[35] Delcourt C, Michel F, Colvez A, Lacroux A, Delage M et al. (2001) Associations of cardiovascular disease and its risk factors with age-related macular degeneration: the POLA study. Ophthal Epidemiol 8: 237-249. doi:10.1076/opep.8.4.237.1613. PubMed: 11471092. 11471092

[36] Wachter A, Sun Y, Dasch B, Krause K, Pauleikhoff D et al. (2004) [Munster age- and retina study (MARS). Association between risk factors for arteriosclerosis and age-related macular degeneration]. Ophthalmologe 101: 50-53. doi:10.1007/s00347-003-0868-1. PubMed: 14872268. 14872268

[37] Nowak M, Swietochowska E, Marek B, Szapska B, Wielkoszynski T et al. (2005) Changes in lipid metabolism in women with age-related macular degeneration. Clin Exp Med 4: 183-187. doi:10.1007/s10238-004-0054-z. PubMed: 15750765. 15750765

[38] Abalain JH, Carre JL, Leglise D, Robinet A, Legall F et al. (2002) Is age-related macular degeneration associated with serum lipoprotein and lipoparticle levels? Clin Chim Acta 326: 97-104. doi:10.1016/S0009-8981(02)00288-7. PubMed: 12417100. 12417100

[39] Tan JS, Mitchell P, Smith W, Wang JJ (2007) Cardiovascular risk factors and the long-term incidence of age-related macular degeneration: the Blue Mountains Eye Study. Ophthalmology 114: 1143-1150. doi:10.1016/j.ophtha.2006.09.033. PubMed: 17275090. 17275090

[40] Delcourt C, Korobelnik JF, Barberger-Gateau P, Delyfer MN, Rougier MB et al. (2010) Nutrition and age-related eye diseases: the Alienor (Antioxydants, Lipides Essentiels, Nutrition et maladies OculaiRes) Study. J Nutr Health Aging 14: 854-861. doi:10.1007/s12603-010-0131-9. PubMed: 21125205. 21125205 PMC3081304

[41] Group CS (2003) Vascular factors and risk of dementia: design of the Three-City Study and baseline characteristics of the study population. Neuroepidemiology 22: 316-325

[42] Bird AC, Bressler NM, Bressler SB, Chisholm IH, Coscas G et al. (1995) An international classification and grading system for age-related maculopathy and age-related macular degeneration. The International ARM Epidemiological Study Group. Surv Ophthalmol 39: 367-374. doi:10.1016/S0039-6257(05)80092-X. PubMed: 7604360. 7604360

[43] Klein R, Klein BE, Knudtson MD, Wong TY, Cotch MF et al. (2006) Prevalence of age-related macular degeneration in 4 racial/ethnic groups in the multi-ethnic study of atherosclerosis. Ophthalmology 113: 373-380. doi:10.1016/j.ophtha.2005.12.013. PubMed: 16513455. 16513455

[44] Lambert JC, Heath S, Even G, Campion D, Sleegers K et al. (2009) Genome-wide association study identifies variants at CLU and CR1 associated with Alzheimer's disease. Nat Genet 41: 1094-1099. doi:10.1038/ng.439. PubMed: 19734903. 19734903

[45] Li Y, Willer CJ, Ding J, Scheet P, Abecasis GR (2010) MaCH: using sequence and genotype data to estimate haplotypes and unobserved genotypes. Genet Epidemiol 34: 816-834. doi:10.1002/gepi.20533. PubMed: 21058334. 21058334 PMC3175618

[46] Li Y, Willer C, Sanna S, Abecasis G (2009) Genotype imputation. Annu Rev Genomics Hum Genet 10: 387-406. doi:10.1146/annurev.genom.9.081307.164242. PubMed: 19715440. 19715440 PMC2925172

[47] 1000 Genomes Project Consortium, Abecasis GR, Altshuler D, Auton A, Brooks LD, Durbin RM, Gibbs RA, Hurles ME, McVean GA (2010) A map of human genome variation from population-scale sequencing. Nature 467: 1061-1073. doi:10.1038/nature09534. PubMed: 20981092. 20981092 PMC3042601

[48] Hartmann D, Thürmann PA, Spitzer V, Schalch W, Manner B et al. (2004) Plasma kinetics of zeaxanthin and 3'-dehydro-lutein after multiple oral doses of synthetic zeaxanthin. Am J Clin Nutr 79: 410-417. PubMed: 14985215. 14985215 10.1093/ajcn/79.3.410

[49] Zeger SL, Liang KY, Albert PS (1988) Models for longitudinal data: a generalized estimating equation approach. Biometrics 44: 1049-1060. doi:10.2307/2531734. PubMed: 3233245. 3233245

[50] Delcourt C, Delyfer MN, Rougier MB, Amouyel P, Colin J et al. (2011) Associations of complement factor H and smoking with early age-related macular degeneration: the ALIENOR study. Invest Ophthalmol Vis Sci 52: 5955-5962. doi:10.1167/iovs.10-6235. PubMed: 21642625. 21642625

[51] Delcourt C, Delyfer MN, Rougier MB, Lambert JC, Amouyel P et al. (2012) ARMS2 A69S Polymorphism and the Risk for Age-Related Maculopathy: The ALIENOR Study. Arch Ophthalmol 130: 1077-1078. doi:10.1001/archophthalmol.2012.420. PubMed: 22893087. 22893087

[52] Tyssandier V, Choubert G, Grolier P, Borel P (2002) Carotenoids, mostly the xanthophylls, exchange between plasma lipoproteins. Int J Vitam Nutr Res 72: 300-308. doi:10.1024/0300-9831.72.5.300. PubMed: 12463105. 12463105

[53] Meyers KJ, Johnson EJ, Bernstein PS, Iyengar SK, Engelman CD et al. (2013) Genetic determinants of macular pigments in women of the carotenoids in age-related eye disease study. Invest Ophthalmol Vis Sci 54: 2333-2345. doi:10.1167/iovs.12-10867. PubMed: 23404124. 23404124 PMC3626525

[54] (2013) Lutein + Zeaxanthin and Omega-3 Fatty Acids for Age-Related Macular Degeneration. The Age-Related Eye Disease Study 2 (AREDS2) Randomized Clinical Trial. JAMA: 1-11 10.1001/jama.2013.4997 23644932

[55] Vingerling JR, Dielemans I, Hofman A, Grobbee DE, Hijmering M et al. (1995) The prevalence of age-related maculopathy in the Rotterdam Study. Ophthalmology 102: 205-210. doi:10.1016/S0161-6420(95)31034-2. PubMed: 7862408. 7862408

